# A case of tractional retinal detachment associated with congenital retinal vascular hypoplasia in the superotemporal quadrant treated by vitreous surgery

**DOI:** 10.1186/s12886-020-01671-y

**Published:** 2020-10-07

**Authors:** Tomomi Miyamoto, Takatoshi Kobayashi, Teruyo Kida, Takaki Sato, Masanori Fukumoto, Tsunehiko Ikeda

**Affiliations:** Department of Ophthalmology, Osaka Medical College, 2-7 Daigaku-machi, Takatsuki City, Osaka, 569-8686 Japan

**Keywords:** Tractional retinal detachment (TRD), Retinal vascular hypoplasia, Optic disc hypoplasia, Macular rotation, Vitrectomy

## Abstract

**Background:**

Here we report a case of traction retinal detachment (TRD) associated with congenital retinal vascular hypoplasia localized in the superotemporal quadrant that was treated with vitrectomy.

**Case presentations:**

A 58 year-old female presented with a gradual decrease of visual acuity (VA) and distorted vision in her left eye. She had a past history of amblyopia in her left eye from early childhood, and a previous examination performed at a nearby hospital revealed that the corrected visual acuity (VA) in that eye was 0.15. Upon initial examination, no abnormal findings were observed in her right eye, yet optic-disc traction and macular rotation with a folded TRD extending superotemporally from the macular region was observed in her left eye. Fluorescein fundus angiography showed a retinal nonperfused area localized in the superotemporal quadrant surrounded by a retinal avascular area. The optic disc in her left eye was smaller than that in her right eye. Vitrectomy was performed to remove the proliferative membrane and created an artificial posterior vitreous detachment (PVD). Following surgery, the patient’s corrected VA improved from 0.04 to 0.1.

**Conclusions:**

The present case was likely to be TRD caused by PVD in the presence of localized congenital retinal vascular hypoplasia secondary to optic-disc hypoplasia.

## Background

Retinal diseases, such as retinopathy of prematurity (ROP) and familial exudative vitreoretinopathy (FEVR), are often accompanied by retinal vascular hypoplasia around the fundus [1, 2] and resultant displacement of retinal blood vessels and the macular region. Although these structures are usually pulled temporally or inferotemporally, a morphology can also be formed depending on the location of the retinal avascular area.

Here we report a case of tractional retinal detachment (TRD) likely caused by age-related progression of posterior vitreous detachment (PVD) that was treated by pars plana vitrectomy (PPV) in a patient with optic-disc traction and macular rotation associated with optic-disc hypoplasia and congenital retinal vascular hypoplasia localized in the superotemporal quadrant.

## Case presentation

A 58-year-old woman presented to our hospital in May 2019 after becoming aware of a gradual decrease of visual acuity (VA) and distorted vision in her left eye. The patient had a history of amblyopia in the left eye from early childhood, and an examination at a nearby hospital in 2016 showed that the VA in that eye was 0.15. She had no history of premature birth, and her family history was unremarkable. Although the patient’s immediate family members, were not directly questioned or clinically examined, examination of both of her sons by a local ophthalmologist revealed no abnormalities.

**Fig. 1:**
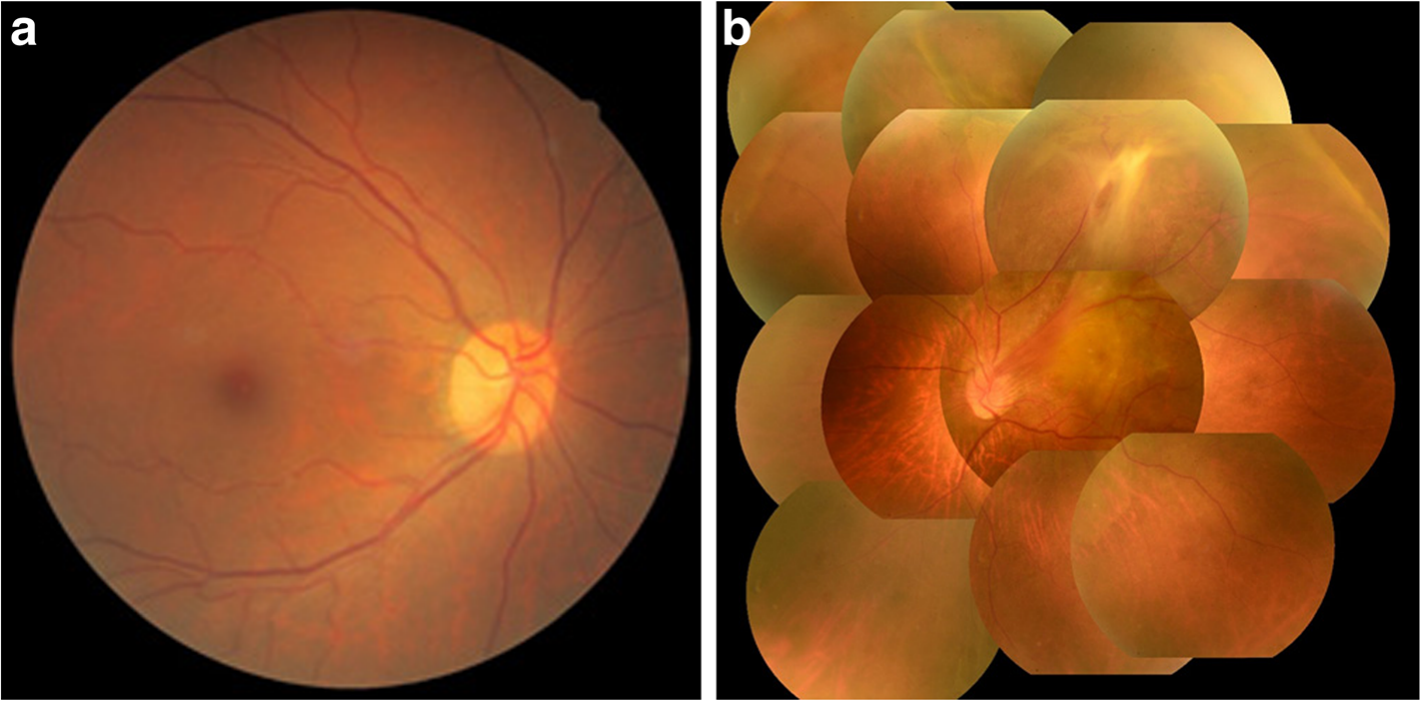
Fundus photographs obtained at the initial examination. Findings revealed no abnormality in the right eye (**a**), yet did reveal a fibroproliferative membrane, macular rotation, and tractional retinal detachment in the superotemporal quadrant in the left eye (**b**)

**Fig. 2:**
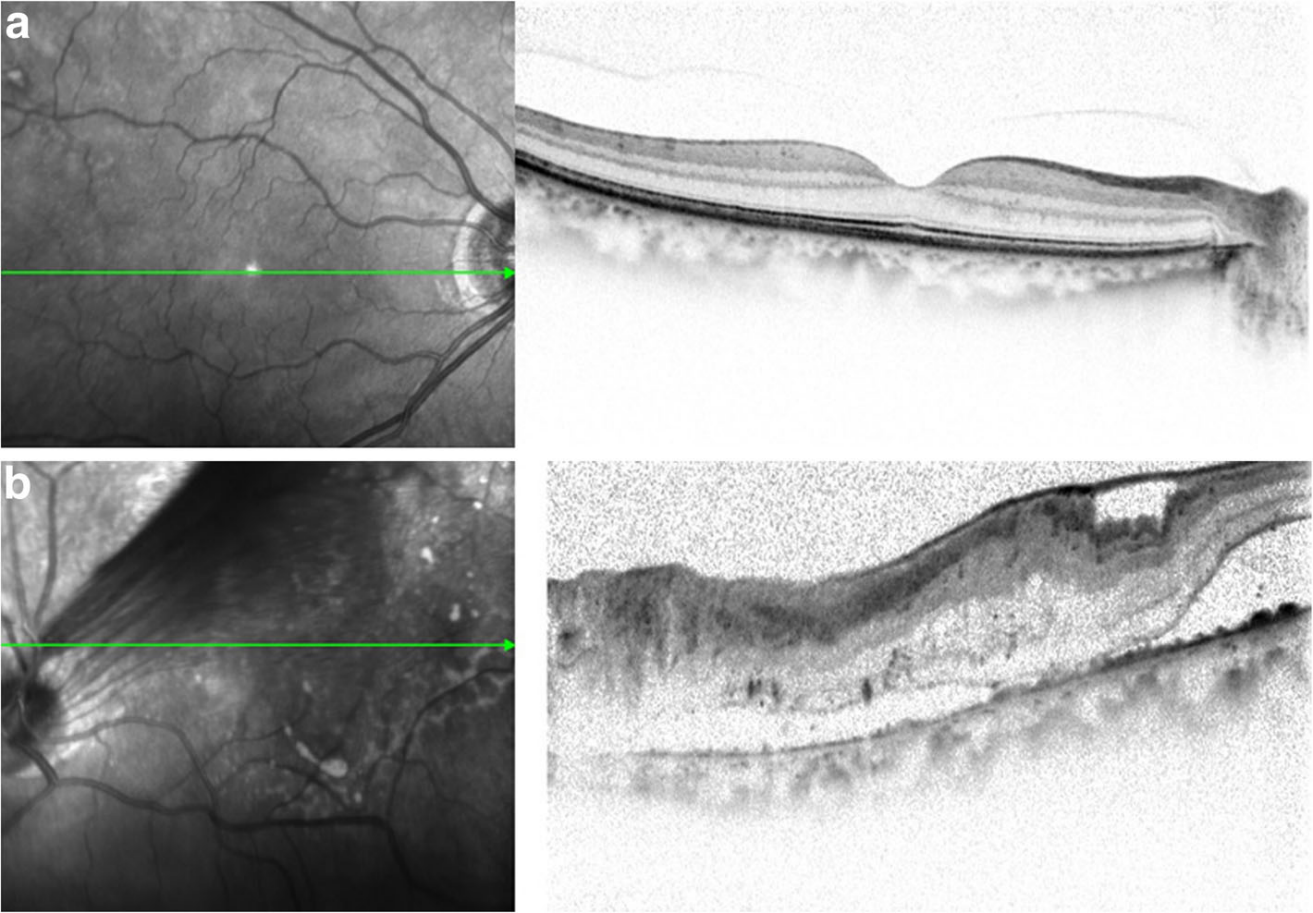
Optical coherence tomography (OCT) imaging obtained at the initial examination. Findings revealed no abnormality in the right eye (**a**), yet did show tractional retinal detachment (TRD) extending into the macular region in the left eye (**b**)

**Fig. 3:**
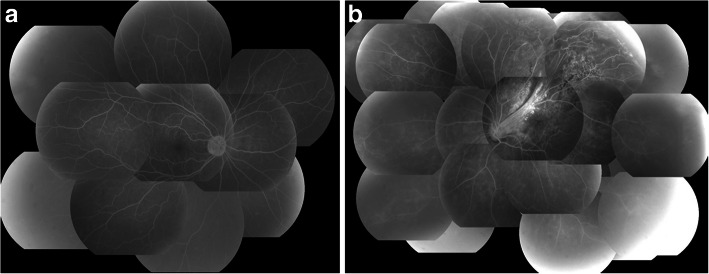
Fluorescein fundus angiography images obtained at the initial examination. Findings revealed no abnormality in the right eye (**a**), yet did show tortuous retinal vessels extending superotemporally surrounded by a focal retinal avascular area in the left eye (**b**)

Upon initial examination, her VA was 0.35 OD (1.0 × S-0.75D:C-1.00D Ax75°) and 0.03 OS (0.04 × C-4.00Ax180°), and her intraocular pressure was 16 mmHg OD and 14 mmHg OS. In both eyes, no abnormalities were observed in the anterior segment, yet examination of the optic media showed a mild cataract. Fundus examination showed no abnormality in the patient’s right eye (Fig. 1a), yet did reveal a fibroproliferative membrane in the superotemporal quadrant and superotemporal macular rotation in the left eye. Moreover, a folded TRD extending superotemporally from the macular region was observed in the left eye (Fig. 1b), and the optic disc in that eye was smaller than that in the right eye. Optical coherence tomography (OCT) (SPECTRALIS**®**, Heidelberg, Engineering GmbH, Heidelberg, Germany) examination showed no abnormalities around the macula in the right eye (Fig. 2a), yet did reveal the TRD extending into the macular region in the left eye (Fig. 2b). In addition, fluorescein fundus angiography revealed no abnormalities in the right eye (Fig. 3a), yet did reveal tortuous retinal vessels extending superotemporally surrounded by a focal retinal avascular area in the left eye. However, the fluorescein leakage from the fibroproliferative membrane in the superotemporal quadrant was not too extensive (Fig. 3b).

**Fig. 4:**
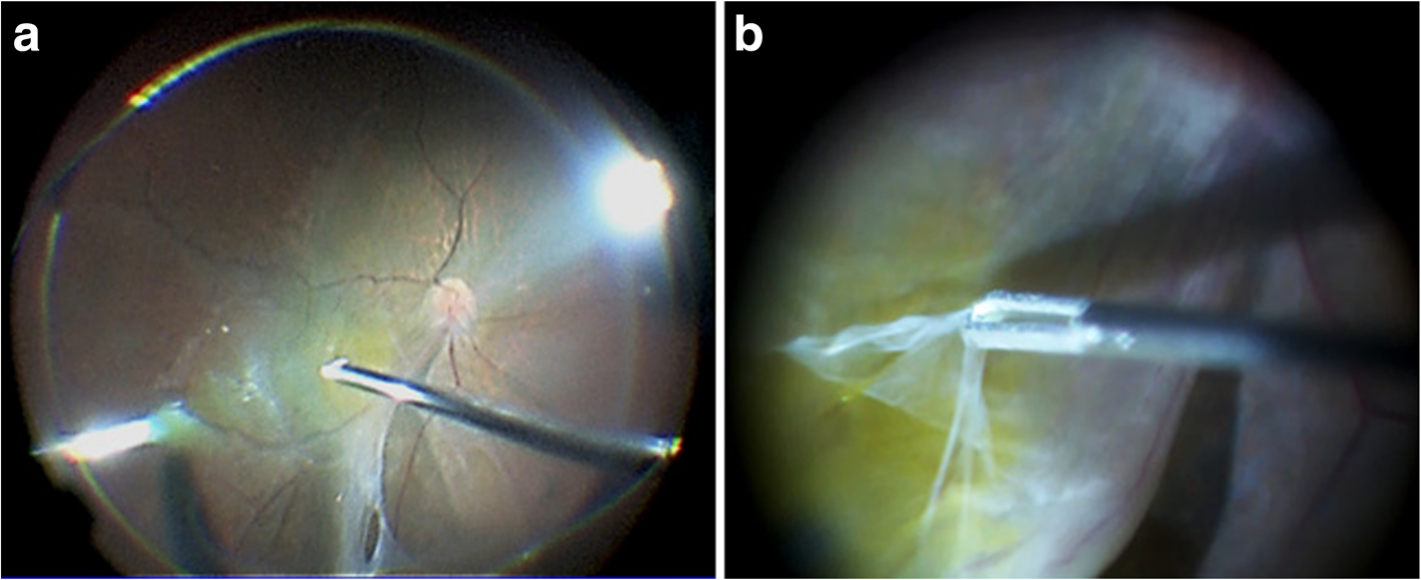
Image of the intraoperative findings in the patient’s left eye. We detached the preretinal membrane from the macular region (**a**) and removed the proliferative membrane connected to the TRD (**b**)

**Fig. 5:**
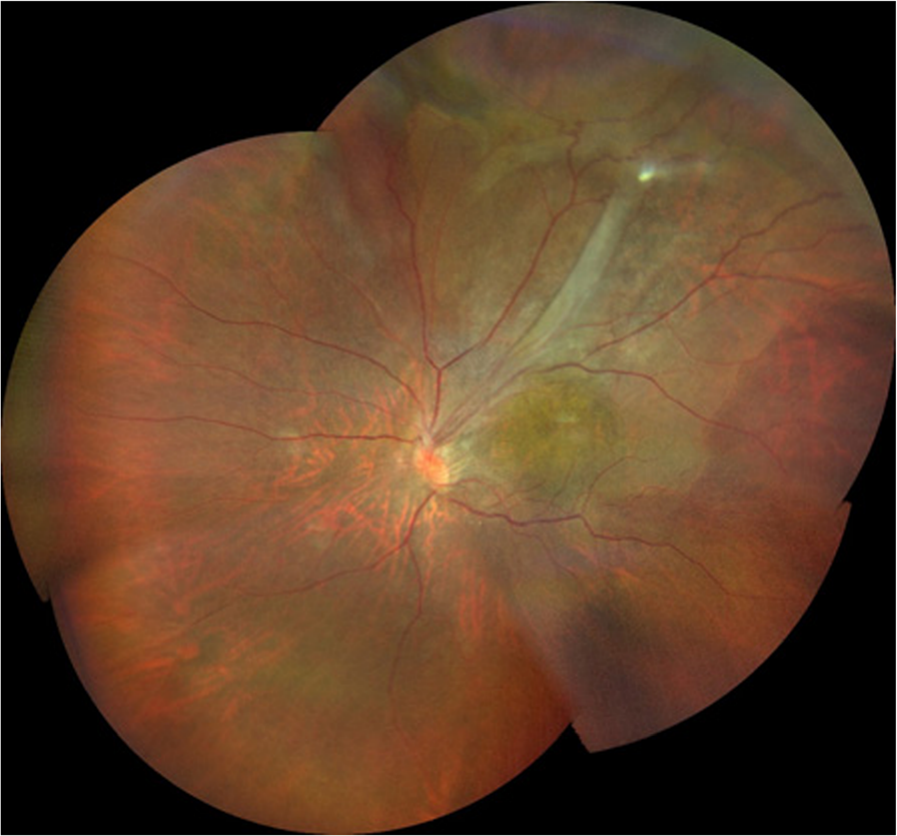
Fundus photographs obtained post vitreous surgery. Post surgery, the subretinal fluid gradually decreased and the e patient’s corrected visual acuity ultimately improved to 0.1

For treatment, phacoemulsification and intraocular lens implantation were performed, followed by PPV to detach the preretinal membrane from the macular region and removal of the proliferative membrane connected to the TRD by use of vitreous forceps (Fig. 4a, b). Triamcinolone acetonide was applied to the posterior pole of the fundus to expose the residual vitreous cortex which was subsequently widely resected using a Diamond Dust Scraper (Synergetics USA Inc., O’Fallon, MO). Next, an artificial PVD was induced toward the periphery, followed by an endophotocoagulation was performed to the non perfusion area. Neither a gas tamponade nor a silicon oil tamponade was performed. Following surgery, an OCT examination revealed that the subretinal fluid had gradually decreased, with the patient’s corrected VA ultimately improving to 0.1 at 6-months postoperative (Fig. 5). The postoperative clinical course involved the administration of, steroid and anti-bacterial eye drops.

## Discussion and conclusion

Diseases that are known to cause peripheral retinal vascular hypoplasia include ROP, FEVR, persistent hyperplastic primary vitreous (PHPV), and incontinentia pigmenti [1–4]. At around 4 months of gestation, retinal blood vessels originate from the optic disc and begin to extend toward the periphery [5]. If their development of the blood vessels is impaired for any reason, a retinal avascular area is formed around the fundus. Since the distance from the optic disc to the ora serrata border is longest on the temporal side, the most extensive retinal avascular area is usually formed on the temporal side, thus resulting in temporal or inferotemporal displacement of the retinal blood vessels and macular region. However, if such impaired vascular development occurs only in one particular quadrant, the retinal vessels and macular region are displaced toward that quadrant, thus causing optic-disc traction and macular rotation. It had been reported that in diseases such as FEVR and PHPV, macular rotation may also occur in the presence of a retinal avascular area or fibroproliferative membrane localized in a particular region [6]. In the present case, it is likely that the formation of a retinal nonperfused area and a retinal avascular area localized in the superotemporal quadrant led to displacement of the retinal blood vessels and macular region toward the lesion, which was further complicated by age-related progression of PVD, ultimately resulting in the progression of TRD.

In regard to the underlying causes, the patient in this study had no history of premature birth or systemic pigmentation anomaly, thus making both ROP and incontinentia pigmenti unlikely causes. Although FEVR is usually bilateral and familial, there have been reports of unilateral cases or cases without a clear family history [7]. Shukla et al. [8] reported mild cases of FEVR that were characterized by the absence of change in the posterior pole, as well as multi-branched/linear retinal vessels in the periphery and avascular areas, which are less obvious findings. Therefore, the possibility of FEVR with a marked difference between right and left could not be ruled out in the present case. PHPV is generally unilateral and non-familial, and is characterized by the presence of a vascularized fibroproliferative membrane in the vitreous cavity, as it involves vitreous vascular abnormality as a major pathogenetic factor. Reportedly, PHPV is also associated with various retinal vascular changes, such as narrowing [9]. Thus, the present case might be an atypical case of PHPV.

It should be noted there have been reported cases of optic-disc hypoplasia and congenital optic-disc abnormality accompanied by retinal avascular areas, fibroproliferative membranes, and/or TRD [10–13]. Shapiro et al. [10] reported 15 cases of optic-disc abnormality accompanied by an area of peripheral retinal nonperfusion and TRD, and Case 7 in their reported series in that study closely resembles the case in this present study. Of the 15 cases in their study, 16 eyes in 9 cases had optic-disc hypoplasia, which was further accompanied by serious retinal nonperfused areas and fibroproliferative membranes in 12 eyes (75%) and TRD in 10 eyes (63%). Moreover, 7 of these patients also had congenital brain diseases, such as septo-optic dysplasia, muscle-eye-brain disease, and lissencephaly. Other similar cases have also been reported [11–13]. For example, in a study by Kiernan et al. [11], the authors reported that in the eyes with a congenital abnormality of the optic disc, the area of peripheral retinal nonperfusion was formed primarily due to impaired development of retinal blood vessels that originated from the optic disc and extended toward the periphery during the fetal period, and that VEGF production from the ischemic retina caused secondary changes, such as fibroproliferative membrane formation and TRD. Although the case in this present study had no obvious systemic disease, the optic disc in the patient’s left eye was smaller than that in the right eye, thus suggesting the involvement of optic-disc hypoplasia in the formation of peripheral retinal vascular hypoplasia. Since there were a limited number of pathways to elucidate the pathology in this present case, we posit that genetic testing may be a valuable tool to help understand the pathology in future cases.

In conclusion, although congenital retinal vascular hypoplasia is rarely accompanied by rapid changes in fundus findings, there is a possibility that age-related progression of PVD can lead to fundus-related changes such as TRD, as observed in the present case. Thus, such cases should be carefully followed-up with regular fundus examinations.

## Data Availability

The datasets during the current study are available from the corresponding author on reasonable request.

## Abbreviations

TRD: Traction retinal detachment
PVD: Posterior vitreous detachment
VA: Visual acuity
ROP: Retinopathy of prematurity
FEVR: Familial exudative vitreoretinopathy
PHPV: Persistent hyperplastic primary vitreous
PPV: Pars plana vitrectomy
OCT: Optical coherence tomography
OD: Oculus dexter
OS: Oculus sinister
